# Endovascular repair of a common femoral artery puncture after losing access using a vascular closure device

**DOI:** 10.1016/j.jvscit.2021.04.026

**Published:** 2021-05-21

**Authors:** Vamsi Reddy, Nathan Todnem, Camila Franco-Mesa, Young Erben, Rabih G. Tawk, Charles Ritchie

**Affiliations:** a School of Medicine, Medical College of Georgia, Augusta, Ga; b Department of Neurosurgery, Baptist Health Medical Group, Louisville, Ky; c Division of Vascular and Endovascular Surgery, Mayo Clinic Florida, Jacksonville, Fla; d Department of Neurological Surgery, Mayo Clinic Florida, Jacksonville, Fla; e Division of Vascular and Interventional Radiology, Mayo Clinic Florida, Jacksonville, Fla

**Keywords:** Endovascular, Common femoral artery, Vascular closure

## Abstract

We present a case of a common femoral artery repair after losing access to the vessel in a 67-year-old woman using a Mynx-Grip vascular closure device. The hematoma over the right common femoral artery was accessed under fluoroscopic guidance. Then, the balloon of the closure device was inflated inside the artery and pulled back against the origin of the puncture site into the common femoral artery. Finally, the collagen plug was deployed and pressed against the outside of the vessel to occlude the puncture site.

Iatrogenic common femoral artery pseudoaneurysm is a common complication and occurs in 0.1% to 0.2% of diagnostic angiograms and 3.5% to 5.5% of interventional procedures.^1^ Although this rate has remained stable, the overall incidence has increased owing to an increase in endovascular procedures within the past decade.^2^ Various surgical and nonsurgical treatment modalities have been used to treat pseudoaneurysms including ultrasound-guided thrombin injection, compression devices and endovascular stent placement.^3^, ^4^, ^5^, ^6^ Herein, we present an alternative technique using a Mynx-Grip vascular closure device (CardinalHealth, Dublin, Ohio) for the management of vessel hemorrhage immediately after the loss of intravascular access and before leaving the endovascular suite to present the formation of the pseudoaneurysm at the access site vessel.^7^

Informed consent was obtained from the patient for this publication.

## Case report

**Fig 1:**
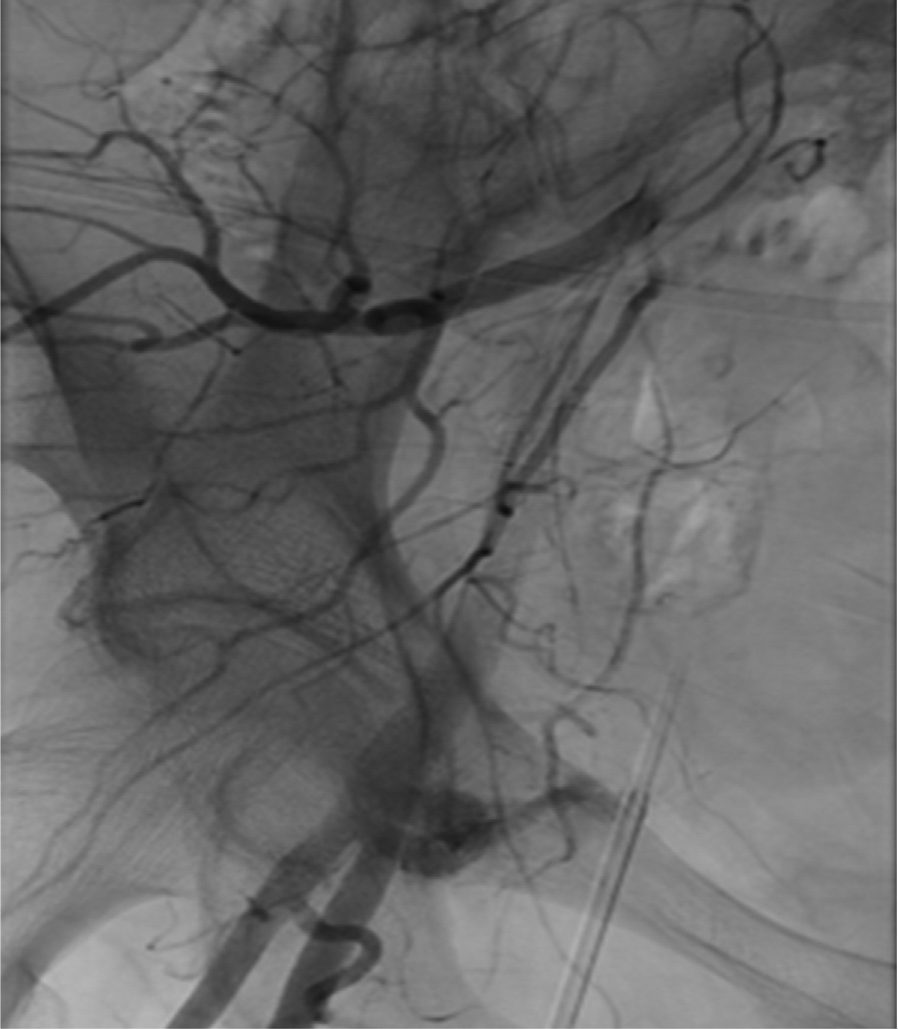
Angiogram after lost access demonstrating a large pseudoaneurysm with contrast extravasation (*White arrow* is the 6F guide sheath adjacent to the artery).

**Fig 2:**
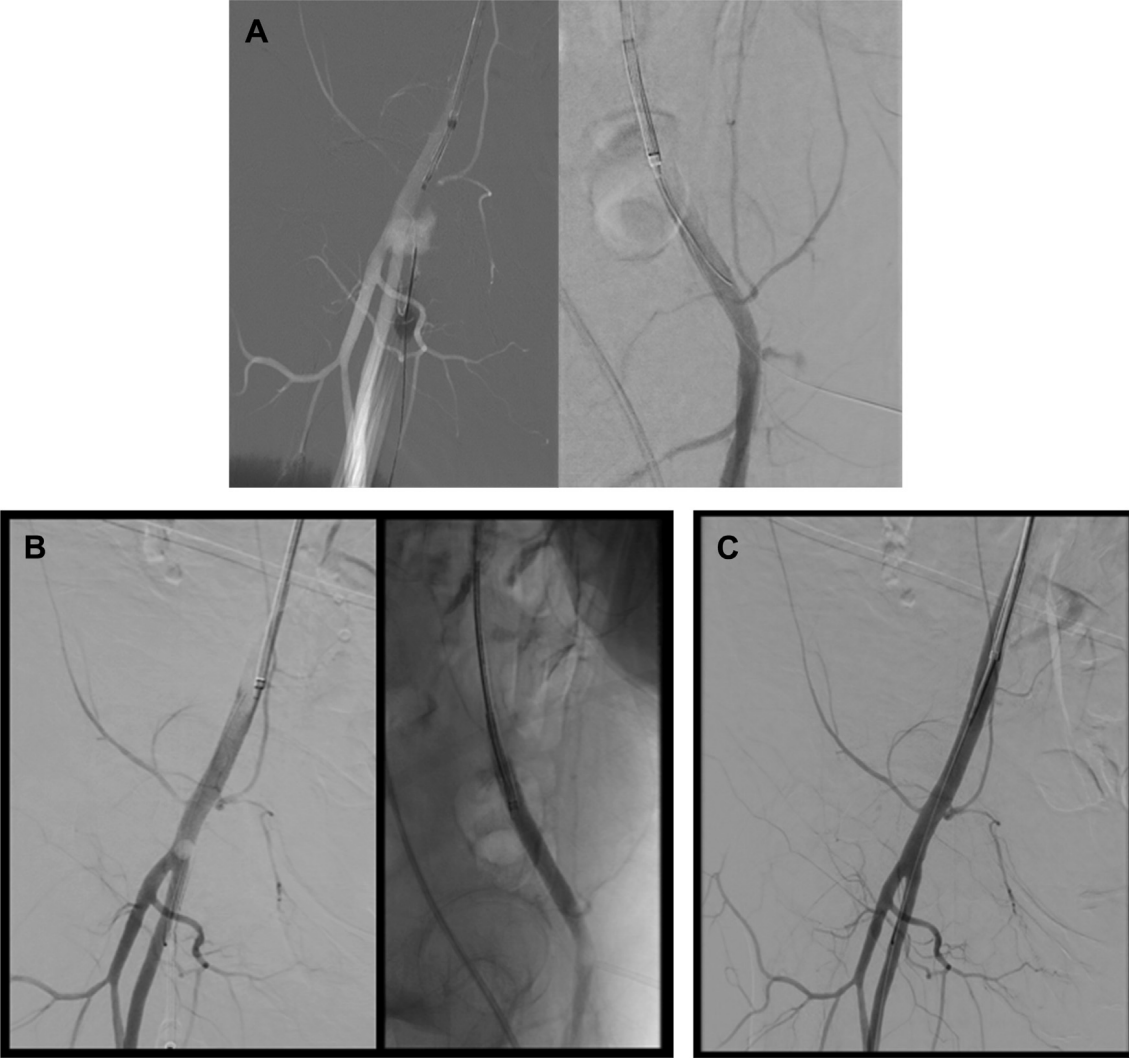
**(A)** Anterior-posterior and lateral views of percutaneous access to the common femoral artery through the pseudoaneurysm with a micropuncture needle and microwire. **(B)** Anterior-posterior and lateral views demonstrating inflation of the Mynx-Grip closure device balloon at the pseudoaneurysm site. **(C)** Final angiogram demonstrating no evidence of residual pseudoaneurysm or contrast extravasation.

A 67-year-old woman presented to our tertiary care center for treatment of a small basilar artery aneurysm. The patient was started on aspirin and clopidogrel for stent assisted coiling of the aneurysm. A 5F 10-cm sheath was inserted into the right common femoral artery. During the exchange from the 5F to a 6F 80-cm guide sheath, access to the right common femoral artery was lost and the 6F guide sheath was dislodged superiorly and medially within the soft tissue. Manual pressure was held over the right common femoral artery access site while new access through the left common femoral artery was obtained. An angiogram of the right common femoral artery demonstrated a large hematoma with contrast extravasation (Fig 1). Despite 40 minutes of manual pressure held over the right common femoral artery, the hemorrhage persisted. The dome of the hematoma was accessed through the original access site under ultrasound and fluoroscopic roadmap guidance using a 21G micropuncture needle and a microwire. A 6F, 10-cm sheath was inserted through the hematoma into the common femoral artery (Fig 2, *A*). Once the sheath location was confirmed within the right common femoral artery, a Mynx-Grip closure device was inserted into the sheath (Fig 2, *B*). The balloon was inflated inside the artery and pulled back against the origin of the puncture and contrast extravasation site. A right femoral artery angiogram was performed demonstrating exclusion of the hemorrhage by the balloon of the closure device. The sheath was pulled out and the collagen plug was deployed and pressed against the outside the vessel to obliterate the puncture site. A final angiogram demonstrated complete obliteration of the puncture site with no evidence of extravasation or vessel dissection (Fig 2, *C*). On follow-up 3 months after the index procedure, the patient does not demonstrate any signs of infection. Duplex ultrasound imaging demonstrates no pseudoaneurysm, dissection, or arteriovenous fistula formation.

## Discussion

The treatment options for iatrogenic pseudoaneurysms are diverse and include a wide range of options from the simple application of pressure over the pseudoaneurysm area to surgical management in complicated cases.^1^ Advances in endovascular technology allowed the development of alternative off-label methods to target vascular repairs in a less invasive manner.^1^ Devices such as the Mynx-Grip typically used for arteriotomy closures^7^^,^^8^ can be safely used in cases in which loss of access has taken place for hemorrhage control and arterial access closure. When Azmoon et al^9^ evaluated the rate of hemostasis after vascular closure after percutaneous coronary intervention with Mynx-Grip devices, vascular complications occurred in 2.1% of 190 patients.^7^ We emphasize in this case report the use of fluoroscopic and ultrasound techniques to access the previously lost arterial access site in adjunctive manner to obtain vascular control.^8^^,^^10^ Other management techniques for the loss of access and hemorrhagic event includes active pressure over the puncture site, placement of a covered stent, prolonged balloon angioplasty, and direct surgical cut-down of the vessel.^11^, ^12^, ^13^ To the best of our knowledge, this alternative closure technique is only possible owing to the dual action of this specific closure device. The intravascular balloon prevents slipping of this device while limiting the hemorrhage; the sealant that is deployed in the outside of the vessel provides the needed agents for thrombosis. There is no similar use of the Mynx-Grip device to repair an arterial puncture complication through access loss by re-accessing the femoral artery through the hematoma and obtaining hemorrhage control using this closure device. This alternative strategy is an appealing option for providers who are familiar with this closure device and should remain in the armamentarium of vascular interventionists.

## Conclusions

This technical case report demonstrates an alternative and effective technique that can be used in the management of iatrogenic arterial access site loss with hemorrhage. Vascular closure devices such as the Mynx-Grip are frequently used with fairly low complication rates. Physicians performing endovascular interventions who are familiar with this closure device can consider this off-label technique to avoid the possible need for stent placement or an open repair of the vessel in question.
